# Laparotomy versus Peritoneal Drainage as Primary Treatment for Surgical Necrotizing Enterocolitis or Spontaneous Intestinal Perforation in Preterm Neonates: A Systematic Review and Meta-Analysis

**DOI:** 10.3390/children10071170

**Published:** 2023-07-06

**Authors:** Gonzalo Solis-Garcia, Agostino Pierro, Bonny Jasani

**Affiliations:** 1 Division of Neonatology, The Hospital for Sick Children, 555 University Avenue, Toronto, ON M5G 1X8, Canada; gonzalo.solis@sickkids.ca; 2 Department of Pediatrics, Mount Sinai Hospital, Toronto, ON M5G 1X5, Canada; 3 Department of Pediatrics, University of Toronto, Toronto, ON M5S 1A1, Canada; agostino.pierro@sickkids.ca; 4 Department of Surgery, The Hospital for Sick Children, Toronto, ON M5G 1X8, Canada

**Keywords:** necrotizing enterocolitis, spontaneous intestinal perforation, laparotomy, peritoneal drainage, neonate

## Abstract

Aim: to systematically review and meta-analyze the impact on morbidity and mortality of peritoneal drainage (PD) compared to laparotomy (LAP) in preterm neonates with surgical NEC (sNEC) or spontaneous intestinal perforation (SIP). Methods: Medical databases were searched until June 2022 for studies comparing PD and LAP as primary surgical treatment of preterm neonates with sNEC or SIP. The primary outcome was survival during hospitalization; predefined secondary outcomes included need for parenteral nutrition at 90 days, time to reach full enteral feeds, need for subsequent laparotomy, duration of hospitalization and complications. Results: Three RCTs (N = 493) and 49 observational studies (N = 19,447) were included. No differences were found in the primary outcome for RCTs, but pooled observational data showed that, compared to LAP, infants with sNEC/SIP who underwent PD had lower survival [48 studies; N = 19,416; RR 0.85; 95% CI 0.79–0.90; GRADE: low]. Observational studies also showed that the subgroup of infants with sNEC had increased survival in the LAP group (30 studies; N = 9370; RR = 0.82; 95% CI 0.72–0.91; GRADE: low). Conclusions: Compared to LAP, PD as primary surgical treatment for sNEC or SIP has similar survival rates when analyzing data from RCTs. PD was associated with lower survival rates in observational studies.

## 1. Introduction

Necrotizing enterocolitis (NEC) is a major contributor to mortality and morbidity in extremely preterm infants [1]. Despite significant improvements in NEC prevention strategies over the last decade [2], its incidence continues to increase worldwide as advances in perinatal care have led to increased survival of extremely preterm infants [3]. Globally, it has been reported that 7% of all very low birth weight (VLBW < 1500 g at birth) infants will suffer an episode of NEC over their neonatal intensive care unit (NICU) hospitalization [3].

Peritoneal drainage (PD), which involves insertion of a Penrose drain into the peritoneum at the bedside, was first reported as a treatment for necrotizing enterocolitis and spontaneous intestinal perforation (SIP) in 1977 by Ein et al. [4], in a case series of five patients who were unstable and would not tolerate laparotomy (LAP). PD aims to decompress the abdomen and remove peritoneal toxic effluents without requiring open surgery, and has established itself as an alternative to LAP, sometimes in hemodynamically unstable infants as a temporizing procedure, but also as a less aggressive and sometimes definitive first line treatment [5]. 

To date, given the overall high mortality [6] and poor neurodevelopmental outcomes [7,8] associated with surgical NEC (sNEC), the first-line surgical treatment approach for sNEC and SIP remains controversial. Previous meta-analysis [9,10] have analyzed data from two published randomized controlled trials (RCTs) [11,12] and numerous observational studies [5] that have compared LAP to PD as primary surgical treatment for NEC and/or SIP, analyzing differences in mortality and other outcomes. Over the last 10 years, however, new evidence, including the largest RCT so far [13] and multiple observational studies have been published, providing new data that might help us answer the question of which is the most appropriate surgical option for preterm neonates with sNEC or SIP. 

The objective of this study was to conduct a systematic review and meta-analysis comparing the mortality and morbidities of LAP and PD when used as primary surgical intervention for sNEC or SIP in preterm neonates. 

## 2. Methods

The design of this study followed the recommendations of PRISMA (Preferred Reporting Items for Systematic Reviews and Meta-analysis) statement [14] and MOOSE (Meta-analysis of Observational Studies in Epidemiology) statement [15]. The protocol was registered in the PROSPERO International Prospective Register of Systematic Reviews database, prior to the start of the literature review (CRD42022302866).

### 2.1. Eligibility of Studies

RCTs and observational studies of preterm neonates who were diagnosed with sNEC or SIP during NICU admission were included. sNEC was defined as NEC requiring surgical intervention according to the different definitions of study authors. Studies were selected when providing data comparing LAP and PD as primary intervention for sNEC or SIP. Studies only including data from one intervention (PD or LAP) were excluded. Only studies with human participants were included in the review, with both full-published articles and conference abstracts evaluated and included if meeting inclusion criteria. Case reports, case series, reviews, letters to editor and commentaries were not included.

### 2.2. Intervention Group

PD was defined as placement of a Penrose drain in the lower abdomen at the bedside in the NICU under local anaesthesia and/or sedation. PD was considered primary intervention when performed as the initial surgical intervention following diagnosis of sNEC or SIP, irrespective of the need for later LAP. 

### 2.3. Comparator

LAP was defined as surgical abdominal exploration with direct observation either in the operating room or at bedside in the NICU.

### 2.4. Outcomes

The primary outcome was survival during hospitalization, and predefined secondary outcomes included need for parenteral nutrition at 90 days, time to reach full enteral feeds, duration of hospitalization and complications including abdominal abscess, intestinal stricture and intestinal fistula. 

### 2.5. Search Strategy 

PubMed, Embase, CINAHL and Cochrane CENTRAL were searched from inception until June 2022. No language restrictions were applied. Searches were conducted by an information specialist, and authors of studies with missing data were contacted via email for additional data or clarification. Reference lists of included studies were also scanned for possible extra studies.

### 2.6. Study Selection

Two authors (GSG and BJ) screened the titles and abstracts independently for potential eligibility, and the same two authors read the subsequent full texts to decide on final inclusion.

### 2.7. Data Extraction

Authors GSG and BJ independently extracted the data with a standardized data collection form which was specifically designed for the study. Potential discrepancies during the data extraction process were resolved by discussion and consensus with the third author (AP).

### 2.8. Risk of Bias 

Two authors (GSG and BJ) conducted risk of bias assessment independently for all the included studies. Cochrane risk of bias tool (ROB) 2.0 [16] was used for RCTs. GSG and BJ independently assessed ROB for the following domains: random number generation, allocation concealment, blinding of intervention and outcome assessors, completeness of follow up, selectivity of reporting and other potential biases. ROB was assigned as low, unclear and high risk based on the Cochrane Collaboration guidelines. The Risk of Bias in Non-randomized Studies of Interventions (ROBINS-I) tool was used for observational studies [17]. Studies were considered low risk of bias only when risk was deemed low for all domains; moderate risk of bias when at least one domain had moderate risk; serious when at least one domain had serious risk; and critical when at least one domain had critical risk of bias. 

### 2.9. Data Synthesis and Analysis

Meta-analysis of pooled data was performed with fixed effects models for RCTs and random effects models for observational studies to account for clinical heterogeneity of included studies, with inverse-variance weighing, following Cochrane Handbook guidelines [18]. Risk ratio (RR) for categorical variables and mean difference (MD) for continuous variables were used as effect measures, both with 95% confidence intervals (CI). Meta-regression was performed to account for heterogeneity and for the possible influence on results of mean gestational age, gestational age differences between study groups, and sample size. Data were analyzed using Review Manager version 5.4.1 and R 4.1.0. 

The heterogeneity of studies was evaluated using the I^2^ statistic and was interpreted according to the Cochrane Handbook guidelines [18]: 0–40%: might not be important; 30–60%: may represent moderate heterogeneity; 50–90%: may represent substantial heterogeneity; 75–100%: considerable heterogeneity. Publication bias was evaluated by funnel plot.

### 2.10. Subgroup Analysis

Pre-defined subgroup analysis was performed for studies including only sNEC patients, for studies including only extremely low birth weight infants (ELBW defined as <1000 g at birth), and for studies published in the last 10 years (2012 to 2022).

### 2.11. Summary of Findings

The most significant information about the magnitude of effect of the intervention, the summary of data available and the certainty of evidence were presented in a “Summary of findings” table that followed the Grading of Recommendations, Assessment, Development and Evaluations (GRADE) guidelines [19]. The certainty of evidence was classified into one of the four categories: high, moderate, low and very low. In case of discrepancies, discussions were held with other two reviewers before reaching consensus.

## 3. Results

**Figure 1:**
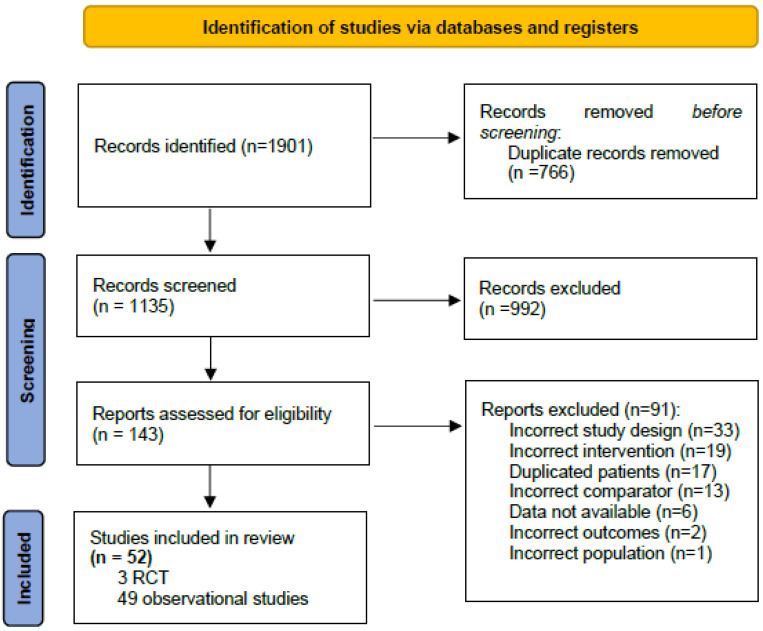
Flow diagram of the literature search and study selection.

The literature search retrieved 1901 potential records which were screened. After removal of 766 duplicates, 1135 records were screened and, finally, 143 full-text articles were assessed for eligibility for inclusion. Finally, 3 RCTs (N = 493 patients) [11,12,13] and 49 observational studies (N = 19,447 patients) [20,21,22,23,24,25,26,27,28,29,30,31,32,33,34,35,36,37,38,39,40,41,42,43,44,45,46,47,48,49,50,51,52,53,54,55,56,57,58,59,60,61,62,63,64,65,66,67,68] were included in the systematic review and meta-analysis. The flow diagram of the literature search and study selection is shown in Figure 1. 

### 3.1. Characteristics of Included Studies

All three RCTs were multicenter and included infants with diagnosis of both SIP or sNEC. The RCT by Blakely et al. [13] reported outcomes for SIP and sNEC separately and the other two studies reporting overall results for the full cohort. Only preterm infants were included in these trials, with the trials by Blakely et al. [13] and Rees et al. [12] including infants <1000 g and Moss et al. [11] including infants <1500 g. Regarding observational studies, most of them (42 studies) were retrospective, with only 5 prospective and 2 ambispective studies. Eleven were multicenter studies, and thirty-eight were conducted in a single center. Thirty studies exclusively included sNEC patients, while eighteen included both NEC and SIP infants and only one study enrolled only SIP patients. The primary outcome was reported in all RCTs and all observational studies except one study by Murcia-Pascual et al. [61]. Supplementary Tables S1 and S2 summarize the characteristics of the included studies (observational studies and randomized controlled trials, respectively).

### 3.2. Risk of Bias Assessment

**Figure 2:**
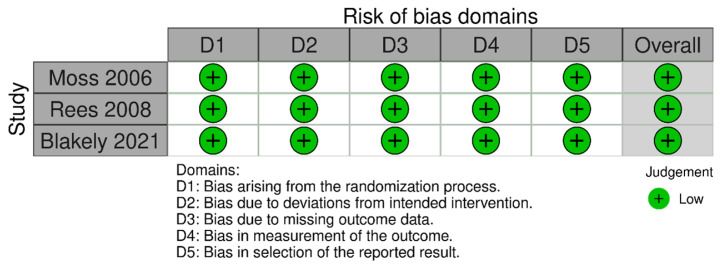
Risk of bias assessment of randomized controlled trials with ROB 2.0 tool [11,12,13].

**Figure 3:**
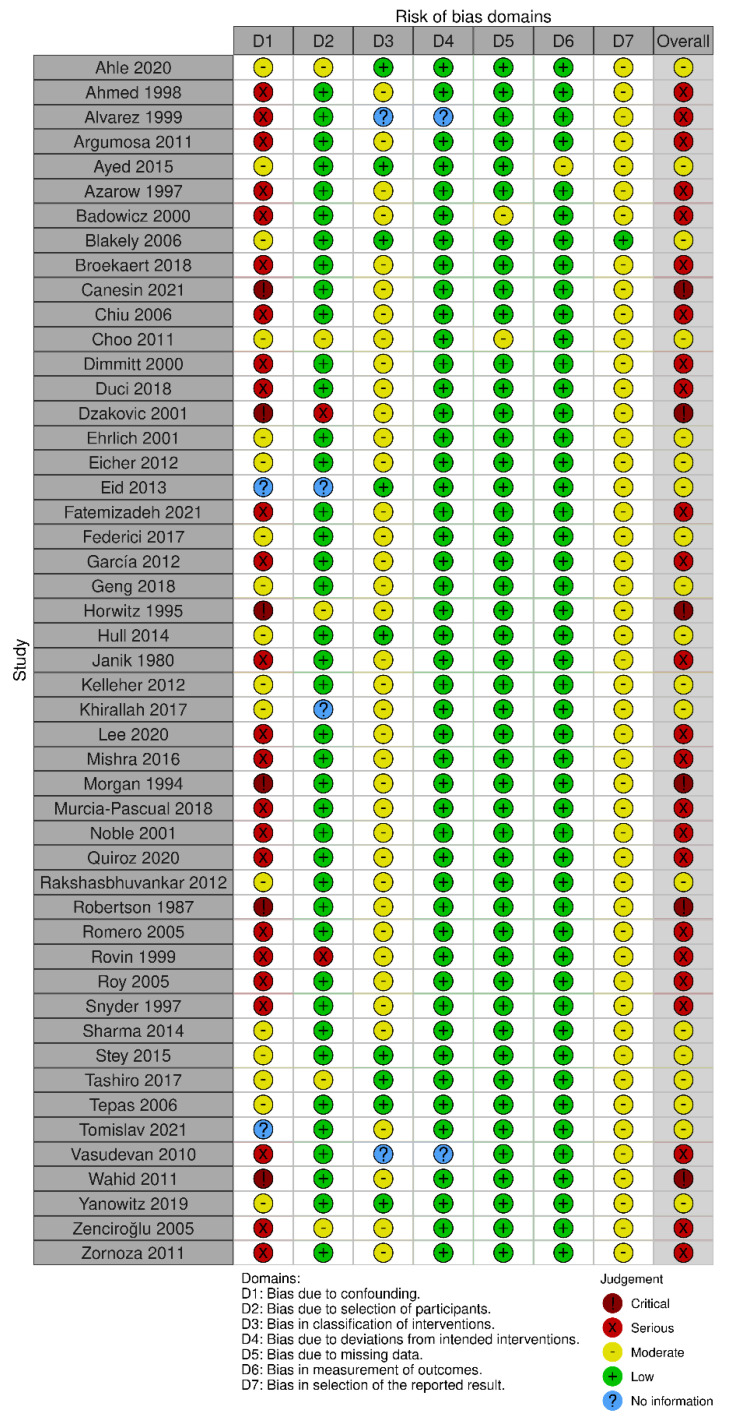
Risk of bias diagram for observational studies via ROBINS-1 [20,21,22,23,24,25,26,27,28,29,30,31,32,33,34,35,36,37,38,39,40,41,42,43,44,45,46,47,48,49,50,51,52,53,54,55,56,57,58,59,60,61,62,63,64,65,66,67,68].

The risk of bias assessment using Cochrane ROB 2.0 tool showed low risk of bias in all domains for RCTs: bias arising from the randomization process, bias due to deviations from intended intervention, bias due to missing outcome data, bias in the measurement of the outcome, and bias in selection of the reported result (Figure 2). Observational studies analyzed with ROBINS-I were found to have moderate to serious risk of bias (Figure 3). Specifically, most observational studies had moderate to serious risk of bias in the domain ‘bias due to confounding’, due to lack of proper adjustment for confounding factors and likely different profile risk between infants receiving laparotomy and peritoneal drainage, having the latter group usually smaller, more immature and sicker infants receiving the intervention. Most of these observational studies also had moderate risk of bias in the domains ‘bias in classification of interventions’ and ‘bias in selection of the reported result’. 

### 3.3. Primary Outcome

**Figure 4:**
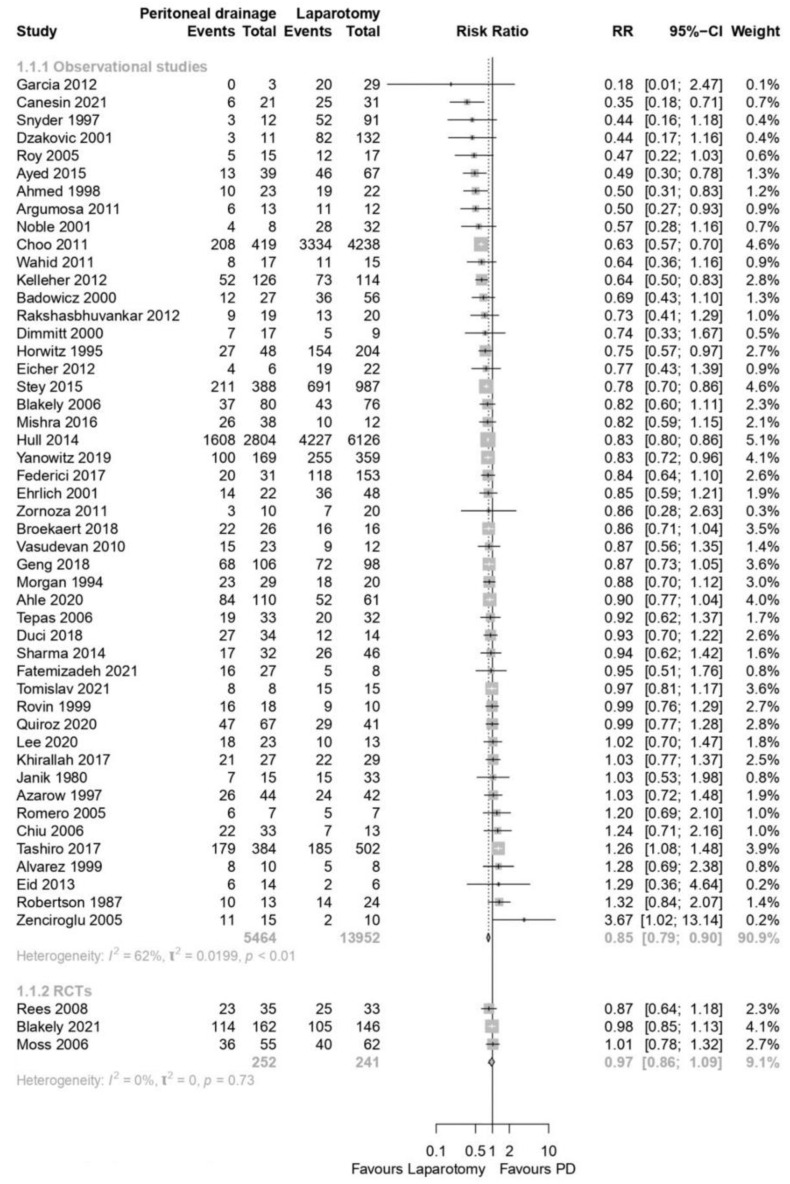
Forest plot for primary outcome: survival during hospital stay [11,12,13,20,21,22,23,24,25,26,27,28,29,30,31,32,33,34,35,36,37,38,39,40,41,42,43,44,45,46,47,48,49,50,51,52,53,54,55,56,57,58,59,60,61,62,63,64,65,66,67,68].

Pooled analyses from 3 RCTs showed no differences in survival between preterm neonates randomized to LAP or PD for sNEC or SIP [3 RCTs; N = 493 patients; Relative risk (RR) 0.97; 95% confidence interval (CI) 0.86–1.09; GRADE: Moderate; Figure 4].

Observational data from 48 studies showed that, compared to LAP, preterm infants with sNEC or SIP who underwent PD had lower survival (48 studies; N = 19,416 patients; RR: 0.85; 95% CI 0.79–0.90; GRADE: low; Figure 4). There was no publication bias, as assessed with the creation of a funnel plot (Supplementary Figure S1).

### 3.4. Secondary Outcomes 

No statistically significant differences were found between the two groups for the different pre-specified secondary outcomes in preterm neonates with sNEC or SIP, including need for TPN at 90 days, time to full feeds, duration of hospital stay and complications, including stricture, abdominal abscess and intestinal fistula (Table 1). These results were similar when including only evidence from RCTs and when including pooled data from observational studies.

### 3.5. Subgroup Analysis

When analyzing studies that only included sNEC infants, pooled observational data showed increased survival in the LAP group (30 studies; N = 9370; RR = 0.82; 95% CI 0.72–0.91; GRADE: low), with only one RCT by Blakely et al. [13] reporting sNEC outcomes separately. This trial did not find significant differences in the primary outcome evaluated in this systematic review, although an increase in survival without neurodevelopmental impairment was found when comparing the two groups using a Bayesian approach to meta-analysis. The study by Moss et al. [11] reported similar results for infants with and without pneumatosis intestinalis. Only one observational study, published by Ahle et al. [63] included exclusively SIP patients, without significant differences in survival between the two groups. These results were similar to the ones reported in the RCT by Blakely et al. [13], in which no significant differences were observed when analyzing the subgroup of infants with SIP. 

For the subgroup of ELBW infants, there were no statistically significant differences for the primary outcome between the two groups when including pooled data from observational studies (16 studies; N = 2072; RR = 0.93; 95% CI 0.81–1.07; GRADE: low), or RCTs (3 studies; 466 patients; RR = 0.96; 95% CI 0.85–1.08; GRADE: Moderate, Supplemental Figure S2). 

Analysis of evidence from observational studies published in the last 10 years (2012–2021) also showed higher survival rates for the LAP group compared to PD (23 studies, N = 13,271, RR = 0.87, 95% CI 0.81–0.94, GRADE: low), with only 1 RCT by Blakely et al. [13] published in the last 10 years and no differences in the primary outcome. 

### 3.6. Meta-Regression

No statistically significant result was obtained when performing meta-regression for an association between survival in PD versus LAP with regards to sample size (coefficient −0.001, *p*-value 0.357), mean gestational age (coefficient −0.01, *p* value 0.59, Supplementary Figure S3) and gestational age differences between study groups (coefficient −0.03, *p*-value 0.12). 

### 3.7. Summary of Findings and Certainty of Evidence 

The certainty of evidence according to the Grading of Recommendations, Assessment, Development, and Evaluations (GRADE) guidelines was graded as low to moderate due to study design and risk of bias. Table 2 (primary outcome) and Supplementary Table S3 (secondary outcomes) summarize certainty of evidence.

## 4. Discussion

In this systematic review and meta-analysis including three RCTs and 49 observational studies for a total of 19,940 preterm infants with sNEC or SIP, we found no differences in survival rates when analyzing high-quality data from RCTs at low risk of bias. When pooling data from observational studies, we found that compared to LAP preterm infants who received PD may have lower survival rates, but this result must be interpreted with caution, given the moderate to serious risk of bias found in the observational studies. The overall certainty of evidence was graded as low to moderate. 

The controversy regarding the ideal first line surgical treatment between LAP and PD for sNEC and SIP in neonates has led to the conduct of three RCTs till date, all of them published in the last two decades. The most recent and largest trial to date is the one published by Blakely et al. [13], whose primary outcome was a composite outcome of death or neurodevelopmental impairment at 18–22 months corrected age, and which included mortality as a secondary outcome. This trial did not find differences for primary and pre-specified secondary outcomes, but in a pre-defined Bayesian sub-analysis of only sNEC patients, PD was found to be associated with an increase in risk of death or moderate-severe cerebral palsy compared to LAP. The previous two trials, published by Moss et al. [11] and Rees et al. [12], did not find statistically significant differences in mortality; however, their statistical methods were frequentist instead of Bayesian, their results were not reported independently for NEC and SIP patients, and both these trials had smaller sample sizes which might have impacted their statistical power to detect differences for key outcomes between the two approaches. The lack of statistical power and the clinical heterogeneity makes it challenging to extract clinically relevant conclusions from the results of the three trials. Although some of the studies and commentaries propose that a better response to the research question may be obtained with larger, adequately powered RCTs, the truth is that those are especially difficult to conduct for surgical interventions in sNEC or SIP in preterm neonatal populations [69], and it is not clear that a larger randomized study might be feasible at the moment. The commonly encountered barriers to designing such trials include difficulties with research ethic board’s approval, challenges with informed consent and low incidence of sNEC and SIP in preterm neonates.

For all these reasons, observational data are a key resource when interpreting evidence in surgical neonatal populations. Unlike RCTs, observational studies are usually able to provide enough statistical power even for small differences, but their design is less robust, as suggested by our analysis, with a significant risk of bias, especially in the confounding domain. Non-randomized studies, in our case and specifically in the PD group, tend to include lower birth weight and more unstable patients who may not be candidates to tolerate a LAP [31,43,66], putting these studies at high risk for residual confounding even after adjustment. With observational data, meta-analysis should always be interpreted with caution due to risk of bias and heterogeneity among included studies [70].

### 4.1. Previous Systematic Reviews and Important Differences from Our Study

The only previous meta-analysis to include a large number of observational studies is the one published by Van Heesewijk et al. [5], which compares the two surgical approaches and evaluated the mortality rates for two RCTs and 25 observational studies published between 1994 and 2016. Similarly to what we describe in this article, they observed higher mortality rates in the PD group on analysis of observational studies and did not find any differences in mortality on pooled analysis from RCTs. The latest meta-analysis comparing the two approaches for sNEC or SIP by Li et al. [71] is the only one published to date that has included the recent trial by Blakely et al. [13]. However, in this review the authors included only 10 observational studies and concluded that no statistically significant differences were found in mortality, which might be explained by a smaller sample size compared to our study, especially given that their confidence interval was of borderline significance, and that they did not include some studies that would have met inclusion criteria as per their description [72].

Our study contributes to this line of investigation by comprehensively including all observational studies published to our knowledge until date, with analysis of a total of 19,940 patients that provides better statistical power compared to previous systematic reviews, and which may explain the difference in the survival between the two surgical approaches for observational data. However, as previously stated, these differences may be more likely to be related to the baseline differences between the study groups, and even though underpowered, the trial data are likely to be more reliable.

As opposed to previous systematic reviews, we also included several secondary outcomes that were not assessed in previous meta-analyses. Similar to findings from previous individual RCTs, our pooled analysis did not show differences in the rates of parenteral nutrition at 90 days, the time to reach full feeds, the duration of hospital stays nor the rates of different complications, which included intestinal strictures, abdominal abscesses and intestinal fistulas. These results were similar for pooled data from RCTs and observational studies. There was significant heterogeneity for most of the outcomes that we meta-analyzed. We explored some possible causes for the heterogeneity of studies with meta-regression techniques, but we did not find that sample size, mean gestational age or gestational age differences between study groups had an impact on the differences between studies.

In addition, our study also analyzed the outcomes between the two surgical approaches for different subgroups, including publications in the contemporary era (2012–2022), ELBW infants and most importantly data for sNEC population alone. As previously stated, this last subgroup of infants is of particular interest, since although sometimes difficult to differentiate in presentation, sNEC and SIP are two clearly separate clinical entities with different pathophysiology and prognosis, and the recent trial by Blakely et al. [13] has suggested that the effect of the two interventions might be different for infants with sNEC but not necessarily for those with SIP. For this subgroup of infants, we found similar results to the overall analysis, with only the trial by Blakely et al. [13] reporting the individual sNEC results and pooled observational data showing increase in survival with LAP, with evidence being low quality due to observational study design and risk of bias. Only the trial by Blakely et al. [13] and the observational study by Ahle et al. [63] reported SIP outcomes independently, without significant differences in survival found in any of these studies but also without overall statistical power to draw meaningful conclusions from their analyses.

The main strengths of our systematic review and meta-analysis are the large sample size, including all RCTs and observational studies comparing the two surgical approaches published until date, comprehensive search of the literature, the inclusion of predefined secondary outcomes and subgroup analysis and the use of meta-regression techniques to explore possible causes of heterogeneity. However, our review also has limitations that must be acknowledged. Firstly, the number of included RCTs is limited to three, and two of them are relatively small, with only 117 [11] and 69 [12] patients, respectively. Secondly, all the observational studies had moderate to serious risk of bias, as shown by our risk of bias analysis via ROBINS-I, which limits the validity of the conclusions drawn from pooled data from these observational studies. Third, long-term data were not reported by most RCTs or observational studies, with only the RCT by Blakely et al. reporting long term outcomes [13]. Fourth, there is a subset of preterm neonates with sNEC or SIP that receive PD followed by LAP due to non-improvement and were not addressed independently in our study. Lastly, a significant number of studies were not able to differentiate between sNEC and SIP, which may present in a similar manner but have different short and long-term prognosis, and in other studies the diagnostic criteria for SIP and NEC were not always clear, making the results of these studies difficult to interpret. Individual data for the two diseases would be needed to draw firm conclusions on the differences in outcomes between the two surgical approaches.

### 4.2. Implications for Clinicians and Researchers

Adequately powered RCT would be the ideal design to confirm these findings, but as previously discussed, they are difficult to conduct due to rarity of sNEC and SIP in preterm neonates. If one were to design a definitive trial, to detect an absolute risk reduction of 10% in mortality (from 40% to 30%) with statistical power of 80% and α error of 0.05, a sample size of 350 ELBW infants per arm would be needed, which would be more than double the size of the biggest trial conducted so far and might be challenging from a logistical perspective even in the setting of a multicenter design. Given the difficulty in designing a trial like this, observational data may be useful; but future studies should include larger sample sizes and aim for balanced study groups, adjusting for possible confounders using multivariable models.

In conclusion, the results of this systematic review and meta-analysis suggest that, compared to LAP, PD as primary intervention for sNEC or SIP may have similar survival rates on analysis of data from clinical trials but may be associated with lower survival rates when pooling data from observational studies.

## Figures and Tables

**Table 1:** Pre-specified secondary outcomes for included RCTs and observational studies.

Outcome	RCTs (N)	Observational (N)	RR or MD for RCTs (CI 95%)	RR or MD for Observational (CI 95%)
TPN at 90 days	2 studies (116)	4 studies (438)	1.18 (0.72 to 1.94)	1.44 (0.95 to 2.18)
Time to full feeds	3 studies (401)	4 studies (324)	7.12 days (−6.91 to 21.15)	4.67 days (−5.46 to 14.80)
Duration of hospital stay	3 studies (395)	12 studies (1961)	11.22 days (−1.56 to 24.00)	6.90 days (−0.13 to 13.94)
Intestinal stricture	3 studies (492)	6 studies (639)	1.42 (0.62 to 3.24)	0.59 (0.27 to 1.31)
Abdominal abscess	3 studies (493)	2 studies (210)	0.80 (0.30 to 2.14)	1.58 (0.51 to 4.90)
Intestinal fistula	2 studies (185)	3 studies (163)	2.99 (0.61 to 14.67)	1.23 (0.59 to 2.57)

TPN: parenteral nutrition; RCTs: randomized controlled trials; RR: relative risk; MD: mean difference; CI: confidence interval.

**Table 2:** Summary of findings for the primary outcome.

Outcome	N	RR or MD	Anticipated Absolute Effects		Quality of Evidence
			With Lap	With PD	
Survival, RCT data	493	RR 0.97 95% CI 0.86 to 1.09	705 infants per 1000 ^1^	684 infants per 1000 (606 to 768 per 1000)	Moderate ^a^
Survival, observational data	19,416	RR 0.85 95% CI 0.79 to 0.90	710 infants per 1000 ^2^	604 infants per 1000 (561 to 639 per 1000)	Low ^b^

^1,2^ Estimate from pooled analysis of control groups. ^a^ Certainty of evidence was deemed moderate in view of the designs of the studies and assessment of risk of bias and downgraded from High given imprecision of results due to limited study sample (two of the studies did not reach pre-specified sample size). ^b^ Certainty of evidence was deemed low due to the design of the studies and the risk of bias.

## Data Availability

Data sets and analysis are available upon request to the corresponding author.

## Supplementary Materials

The following supporting information can be downloaded at: https://www.mdpi.com/article/10.3390/children10071170/s1, Table S1. Characteristics of included observational studies; Table S2. Characteristics of included RCTs; Figure S1. Funnel plot evaluating publication bias; Figure S2. Forest plot for primary outcome, subgroup analysis of infants <1000 g; Figure S3. Bubble plot for gestational age metaregression; Table S3. Summary of findings, secondary outcomes.
