# Supplementary material for: Laparotomy versus Peritoneal Drainage as Primary Treatment for Surgical Necrotizing Enterocolitis or Spontaneous Intestinal Perforation in Preterm Neonates: A Systematic Review and Meta-Analysis
Source: Children (Basel). 2023 Jul 6;10(7):1170. doi: 10.3390/children10071170 (PMC10378122; doi:10.3390/children10071170)
Supplement: Supplementary file 1 [file children-10-01170-s001.zip › children-2370772-supplementary.pdf]

Laparotomy versus Peritoneal Drainage as Primary Treatment for Necrotizing Enterocolitis or Spontaneous Intestinal Perforation in Neonates: A Systematic Review and Meta-analysis

Online supplementary material

Contents	Page
Supplementary Table S1. Characteristics of included observational studies	2-3
Supplementary Table S2. Characteristics of included RCTs	4
Supplementary Figure S1: Funnel plot evaluating publication bias.	5
Supplementary Figure S2. Forest plot for primary outcome, subgroup analysis of infants <1000g.	6
Supplementary Figure S3. Bubble plot for gestational age metaregression	7
Supplementary Table S3. Summary of findings, secondary outcomes.	8

Supplementary Table S1: Characteristics of included observational studies

Author, year	N	Design	Diagnosis	GA
Ahle 2020 [63]	171	Multicentre, retrospective	SIP	25.7 (2.4)*
Ahmed 1998 [26]	45	Single center, retrospective	NEC	30.9 (3.3)*
Alvarez 1999 [27]	18	Single center, retrospective	NEC	31
Argumosa 2011 [41]	25	Single center, retrospective	NEC, SIP	26.5 (23-33)**
Ayed 2015 [52]	106	Multicentre, retrospective	NEC	25.8 (1.4)*
Azarow 1997 [24]	86	Single center, retrospective	NEC, SIP	29.5
Badowicz 2000 [29]	83	Multicentre, retrospective	NEC	33.3 (4.1)*
Blakely 2006 [37]	156	Multicentre, retrospective	NEC, SIP	25.2 (22-31)**
Broekaert 2018 [58]	42	Single center, retrospective	NEC, SIP	24.3 (23.2-25.6)***
Canesin 2021 [66]	52	Single center, retrospective	NEC	31.0 (2.9)*
Chiu 2006 [38]	46	Single center, retrospective	NEC, SIP	27.9
Choo 2011 [42]	4657	Multicentre, retrospective	NEC	NR
Dimmitt 2000 [30]	26	Single center, retrospective	NEC	25.5 (1.7)*
Duci 2018 [59]	48	Single center, retrospective	NEC, SIP	26 (23–34.9)**
Dzakovic 2001 [31]	143	Single center, retrospective	NEC	27 (0.59)*
Ehrlich 2001 [32]	70	Single center, retrospective	NEC	NR
Eicher 2012 [45]	28	Single center, retrospective	NEC, SIP	24.3 (24-26)***
Eid 2013 [49]	20	Single center, prospective	NEC	NR
Fatemizadeh 2021 [67]	36	Single center, prospective	SIP	25.3
Federici 2017 [55]	184	Multicentre, retrospective	NEC	NR
García 2012 [46]	32	Single center, ambispective	NEC	33.9 (27-41)**
Geng 2018 [60]	294	Multicentre, retrospective	NEC	28.0 (2.0)*
Horwitz 1995 [23]	252	Single center, retrospective	NEC	31 (5.0)*
Hull 2014 [50]	8935	Multicentre, prospective	NEC, SIP	25.9 (2.3)*
Janik 1980 [20]	48	Single center, retrospective	NEC	30 (25-39)**
Kelleher 2012 [47]	240	Single center, retrospective	NEC	26.5 (25-31)**

Khirallah 2017 [57]	56	Single center, prospective	NEC	31.9
Lee 2020 [64]	36	Single center, retrospective	NEC, SIP	25.4 (23-39)**
Mishra 2016 [54]	50	Single center, retrospective	NEC, SIP	36 (26-42)**
Morgan 1994 [22]	49	Single center, retrospective	NEC	29.0 (0.7)*
Murcia-Pascual 2018 [61]	31	Single center, retrospective	NEC	27.3 (1.3)*
Noble 2001 [33]	40	Single center, retrospective	NEC, SIP	26.4
Quiroz 2020 [65]	108	Single center, ambispective	NEC, SIP	27.5 (3.6)*
Rakshasbhuvankar 2012 [48]	39	Single center, retrospective	NEC, SIP	25.0 (23.2-29.8)**
Robertson 1987 [21]	37	Single center, retrospective	NEC	NR
Romero 2005 [34]	14	Single center, retrospective	NEC	29.1 (3.8)*
Rovin 1999 [28]	28	Single center, retrospective	NEC, SIP	28.2 (3.1)*
Roy 2005 [35]	32	Single center, retrospective	NEC	32.5
Sharma 2014 [51]	78	Single center, prospective	NEC, SIP	26.5 (2.2)*
Snyder 1997 [25]	103	Single center, retrospective	NEC	31.1
Stey 2015 [53]	1375	Multicentre, retrospective	NEC	27 (25-33)***
Tashiro 2017 [56]	886	Multicentre, retrospective	NEC	23-34**
Tepas 2006 [39]	65	Single center, retrospective	NEC, SIP	25.5 (1.7)*
Tomislav 2021 [68]	23	Single center, retrospective	NEC	27.7 (3.7)*
Vasudevan 2010 [40]	35	Single center, retrospective	NEC, SIP	25.7 (1.9)*
Wahid 2011 [43]	32	Single center, retrospective	NEC	25.2
Yanowitz 2019 [62]	528	Multicentre, retrospective	NEC	25 (23-26)***
Zenciroğlu 2005 [36]	25	Single center, retrospective	NEC	32.1 (3.8)*
Zornoza 2011 [44]	30	Single center, retrospective	NEC	26.1 (2.1)*

GA: gestational age. NEC: necrotizing enterocolitis. SIP: spontaneous intestinal perforation. *Mean and SD **Median and range. ***Median and interquartile range. NR: not reported. GA is reported as median in studies reporting range or IQR, and as mean otherwise.

Supplementary Table S2: Characteristics of included RCTs

Study	N	Design	Inclusion criteria	GA
Moss 2006 [11]	117	Multicentre RCT	SIP/NEC <1500g	26 (2.1)
Rees 2008 [12]	69	Multicentre RCT	SIP/ NEC <1000g	26.3 (0.3)
Blakely 2021 [13]	310	Multicentre RCT	SIP/ NEC <1000g	25 (1.7)

RCT: randomized controlled trial. GA: gestational age. NEC: necrotizing enterocolitis. SIP: spontaneous intestinal perforation. GA values expressed as mean (SD)

Supplementary Figure S1: Funnel plot evaluating publication bias

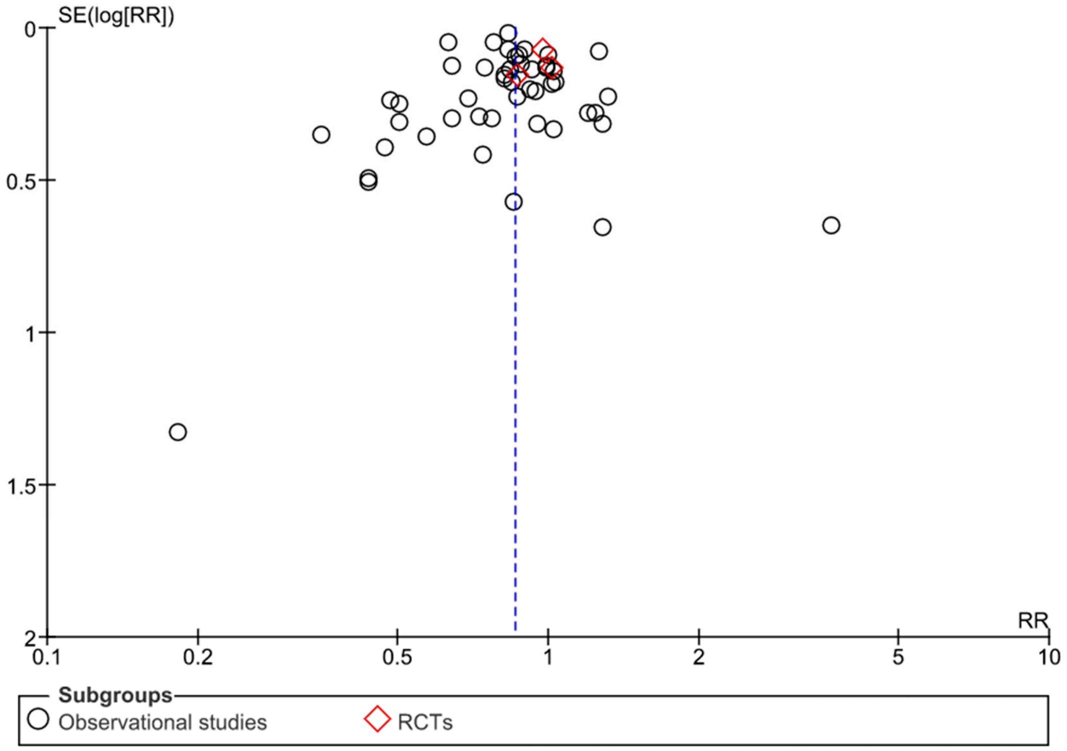

Supplementary Figure S2: subgroup analysis of infants <1000g

Study	Peritoneal drainage Events	Peritoneal drainage Total	Laparotomy Events	Laparotomy Total	RR	95%-CI	Weight
**1.2.1 Observational studies**							
Azarow 1997	20	29	2	9	3.10	[0.89; 10.79]	0.6%
Badowicz 2000	3	13	4	6	0.35	[0.11; 1.08]	0.7%
Blakely 2006	37	80	43	76	0.82	[0.60; 1.11]	6.7%
Broekaert 2018	22	26	16	16	0.86	[0.71; 1.04]	11.0%
Dimmitt 2000	7	17	5	9	0.74	[0.33; 1.67]	1.4%
Ehrlich 2001	14	22	36	48	0.85	[0.59; 1.21]	5.5%
Eicher 2012	4	6	19	22	0.77	[0.43; 1.39]	2.5%
Fatemizadeh 2021	16	27	5	8	0.95	[0.51; 1.76]	2.3%
Horwitz 1995	12	22	32	55	0.94	[0.60; 1.46]	4.0%
Janik 1980	4	8	1	6	3.00	[0.44; 20.44]	0.3%
Lee 2020	18	23	10	13	1.02	[0.70; 1.47]	5.3%
Morgan 1994	12	17	3	5	1.18	[0.54; 2.56]	1.5%
Tashiro 2017	179	384	185	502	1.26	[1.08; 1.48]	12.5%
Tepas 2006	19	33	20	32	0.92	[0.62; 1.37]	4.7%
Yanowitz 2019	100	169	255	359	0.83	[0.72; 0.96]	13.2%
Zornoza 2011	3	10	7	20	0.86	[0.28; 2.63]	0.8%
		886		1186	0.93	[0.81; 1.07]	73.2%
Heterogeneity: $I^2 = 49\%$, $\tau^2 = 0.0278$, $p = 0.01$							
**1.2.2 RCTs**							
Blakely 2021	114	162	105	146	0.98	[0.85; 1.13]	13.2%
Moss 2006	29	45	30	45	0.97	[0.72; 1.30]	6.9%
Rees 2008	23	35	25	33	0.87	[0.64; 1.18]	6.7%
		242		224	0.96	[0.85; 1.08]	26.8%
Heterogeneity: $I^2 = 0\%$, $\tau^2 = 0$, $p = 0.78$							

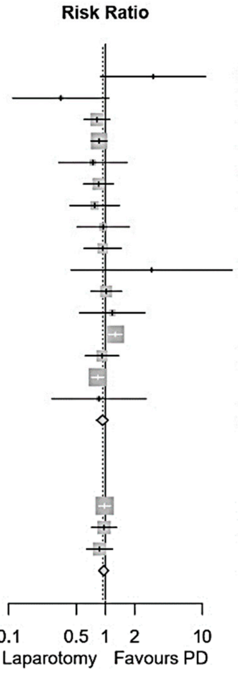

Supplementary Figure S3: Bubble plot for gestational age meta-regression

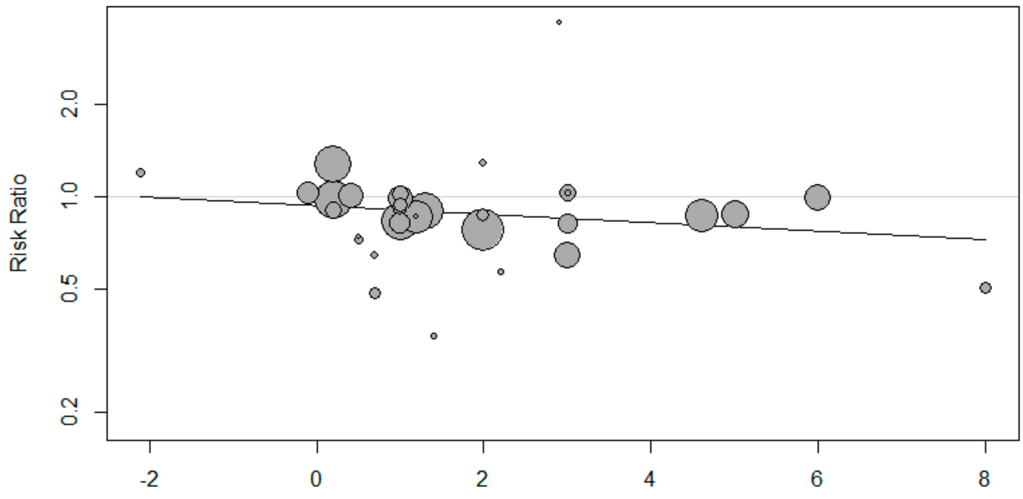

Linear regression of RR by differences in GA between study groups. Coefficient -0.03, p value 0.12.

Supplementary Table S3: Summary of findings for pre-specified secondary outcomes

Outcome	N	RR or MD	Anticipated absolute effects		Certainty of evidence
			With Lap	With PD	
TPN at 90 days, RCTs	116	RR 1.18<br>95% CI 0.72 to 1.94	181 infants per 1000[1]	214 infants per 1000<br>(130 to 351 per 1000)	Moderate[a]
TPN at 90 days, observational studies	438	RR 1.44<br>95% CI 0.95 to 2.18	130 infants per 1000[1]	187 infants per 1000<br>(124 to 283 per 1000)	Low[a]
Time to reach full enteral feeds, RCTs	401	MD 7.12 days<br>95% CI -6.91 to 21.15	49 days[1]	56 days<br>(42 days to 70 days)	Moderate[a]
Tim to reach full enteral feeds, observational studies	324	MD 4.67 days<br>95% CI -5.46 to 14.80	30 days[1]	35 days<br>(25 days to 45 days)	Very Low[a]
Duration of hospital stay, RCTs	395	MD 11.22 days<br>95% CI -1.56 to 24.00	112 days[1]	123 days<br>(110 days to 136 days)	Moderate[a]
Duration of hospital stay, observational studies	1961	MD 6.90<br>95% CI -0.13 to 13.94	93 days[1]	100 days<br>(93 days to 107 days)	Low[a]
Intestinal stricture, RCTs	492	RR 1.42<br>95% CI 0.62 to 3.24	38 infants per 1000[1]	54 infants per 1000<br>(24 to 123 per 1000)	Moderate[a]
Intestinal stricture, observational studies	639	RR 0.59<br>95% CI 0.27 to 1.31	140 infants per 1000[1]	83 infants per 1000<br>(38 to 183 per 1000)	Low[a]
Abdominal abscess, RCTs	493	RR 0.80<br>95% CI 0.30 to 2.14	33 infants per 1000[1]	26 infants per 1000<br>(10 to 71 per 1000)	Moderate[a]
Abdominal abscess, observational studies	210	RR 1.58<br>95% CI 0.51 to 4.90	50 infants per 1000[1]	79 infants per 1000<br>(26 to 245 per 1000)	Very Low[a]
Intestinal fistula, RCTs	185	RR 2.99<br>95% CI 0.61 to 14.67	21 infants per 1000[1]	63 infants per 1000<br>(13 to 308 per 1000)	Moderate[a]
Intestinal fistula, observational studies	163	RR 1.23<br>95% CI 0.59 to 2.57	130 infants per 1000[1]	160 infants per 1000<br>(77 to 334 per 1000)	Low[a]

[1]Estimate from pooled analysis of control groups. [a]Certainty was deemed Moderate in view of study design, risk of bias, downgraded from High given imprecision of results due to limited study sample. [b]Certainty was deemed low in view of the study design and risk of bias
